# Proceedings of the Illinois State Medical Society

**Published:** 1851-11

**Authors:** 


### Part 2—Reviews and Notices of New Works

### ARTICLE I.

*Proceedings of the Illinois State Medical Society at its first Annual Meeting held in Peoria, June 3d,* 1851. *Published under the supervision of the Committee on Publication.* Chicago, JAS. J. LANGDON, Book and Job Printer, 1851. pp. 59.

We are in receipt of a copy of this document. It has been late in making its appearance,partly as we understand owing to the delay in gathering together the papers, from different individuals, of which it is principally composed. We could have pardoned a much longer delay, however, if it would have secured more accuracy in the printing.

There are some mistakes of rather an amusing character. In Dr. Thompson's report, it is said an Autopsical examination of the brain found "the pea-water engorged."

The first twenty-two pages of the document are occupied by the minutes of the proceedings of the Society and Prof. Herrick's Address, both of which have been reported in the Journal. The remaining portion is made up of three papers, viz: Chloroform in Surgical operations, by E. S. Cooper, M. D., The Report of the Committee on Practical Medicine and a partial Report from the Committee on Obsteterics, by R. Rouse, M. D., after which follows a list of the members of the Society.

The paper of Dr. Cooper says, the author "has had an opportunity of testing the effects of Chloroform as an æesthetic agent in seventy-nine cases of Surgical operations since the organization of the State Medical Society. Most of these, of minor importance, so far as the operations are concerned, but displaying no less, as the writer thinks, the merits of the article itself in producing insensibility."

The only remarkable case among these was one which is reported, in which the rapid inhalation of Chloroform produced prostration, suffocation, and asphyxia which only gave way by the most active stimulation.

A subsequent attempt proved that the Chloroform could be safely used in the case by commencing its inhalation gradually. The case is instructive and worthy of note.

The Report on Practical Medicine by Dr. Samuel Thompson, Chairman, first discusses the subject of Intermittant and Remittent Fevers. The difficulty of defining any of these as congestive Fever is clearly pointed out, and the terms Malignant Remittent or Intermittent proposed as a substitute.

The prevalence of a form of disease called Typhus or Typhoid Fever within the last few years, in different parts of Illinois, is noticed, but the reporter is not able clearly to make out its nosological character.

The disease called Milk Sickness receives the attention it deserves. But we should incline to differ from the author who calls it a "truly epidemic disease," as our own observations and the history we have read of its prevalence would compel us to call it strictly endemic in its character. It is by the showing of the report itself, confined to circumscribed localities.

The author of the report, whom our readers will recollect published a long and exceedingly well written article upon the subject of Malaria in the number of this Journal for May, 1850, seems to have contracted a Miasmataphobia as he attributes to it the production of many of the diseases noticed, among which may be mentioned, not only the Fevers of the West, but Milk Sickness and Asiatic Cholera. In the article above referred to the position is taken in reference to the Milk

Sickness and it is re-asserted in the report before us. The treatment of the malady is discussed at length with reports of the practice of various practitioners.

The conclusion to which the author arrives is that Bicarb. Soda freely given with a little Sulpharic Acid to form an efferversing draught and also a Sulphate of Soda to act as a cathartic, with Quinine and Calomel are the principle remedies of importance. Tonics in the latter stage of the disease are recommended.

The course of treatment for this disease that we found safest and the most speedily successful, was suggested to us by Dr. N. Wilson now of Iroquois Co. Ills, who had acquired an extensive reputation for the success of his treatment of Milk Sickness on the Wabash River in Indiana. The most prominent symptoms of the disease are the nausea and vomiting and the obstinate constipation, the former of which are much in the way of the use of remedies for relieving the latter, which in turn seems to keep up the sickness; as catharsis usually brings relief. The treatment referred to was, to select some mild innocous cathartic, as equal parts of Pulv. Rheubarb and calcined Magnesia which Dr. W. prefered, and repeat the dose of it as often as rejected by the stomach, or at least at short intervals, until free catharsis was secured, when the danger is generally overcome. After this some mild tonics with a sufficiently free use of laxatives to prevent a return of coprostasis, completed the cure. The use of Calomel was early abandoned, on account of the danger of producing ptyalism from its remaining too long in the system before operating, and the conviction that any other cathartic that could be retained so as to act, was equally efficacious. Relapses, to which patients are exceedingly liable from much exercise after an attack of the disease, may be prevented by attention to keeping the bowels open regularly, which should always be strictly enjoined in discharging convalescents from this malady.

After some remarks upon Pneumonitis, Scarlatina, Rubeola, and the use of Iodine injections in dropsical affection, by Prof. Brainard, the report takes up the subject of Cholera and devotes the remaining twelve pages to its consideration. But little is said of its treatment, and nothing seems to have thrown new light upon this dark part of the subject.

The cause of the disease claims the principal share of attention, and singularly enough in a document professing to be a report of what has transpired, the author has compiled a most imperfect history of the appearance and progress of the disease, selecting mostly such parts of the written history, as give plausibility to his own peculiar views of the origin of the disease, or arguments against those entertained by others, italicising the phrases and sentences that are designed to bear upon the questions at issue.

He first undertakes to disprove the contagiousness of Cholera and subsequently to establish its malarious origin. His success and means of accomplishing these undertakings we will endeavor to make apparent.

In giving a history of its introduction into Illinois, the author refers to its appearance at Quincy and Galena early in the spring of 1849, where the reporter says in italics, "it did not appear to spread," omitting to notice the fact that it was brought to Galena by emigrants from the lower Mississippi where the disease was at the time prevailing.

In giving the history of its appearance in Chicago, the author gives credit to the editor of this Journal for his facts, but singularly enough asserts that "it did not appear to spread much in Chicago till the 5th of June." My statement is, that "the disease had prevailed in other parts of the city for two months before this (the Norwegian) neighborhood, (where it prevailed with the greatest mortality) was affected." We would gladly omit this correction but justice to the cause of truth seems to demand that history shall not be mis-quoted

That the Cholera has generally, as in Chicago, prevailed more extensively in hot weather than in cold, is a fact so universally admitted that it was not necessary to mis-state the facts of the case to make it appear ; nor even to put the phrase "till the heat of July had set in," in italics.

It is very singular that the reporter should have made no mention at all of the introduction of Cholera into the numerous isolated settlements in the State, the history of which in a large number of instances, well authenticated by numerous references, was before him. The only explanation of this omission that appears plausible is that they furnish no evidence of the Malarious origin of the disease and most clearly and forcibly corroborate the doctrine of its communicable nature.

The arguments of the report drawn from the coincidence of the prevalence and origin of Cholera in several places where "the atmosphere was humid, murky, close and oppressive," and in some marshy and malarious places, and his showing that high, dry and sandy places might be sources of malaria from the vegetable products being washed into the substratum to putrify, are fair specimens of the arguments generally urged against the doctrine of contagion.

The first is deficient in not being by any means a uniform attendant upon the prevalence of the disease as its fearful mortality in Norway and Sweeden during the intense cold of winter, and many other instances in its history prove ; and the second affords no explanation of its frequently sparing marshy and generally unhealthy districts, and often affecting otherwise the most healthy spots on the face of the earth.

Great stress is laid upon the fact, that exposure to Cholera does not always propagate the disease, which may be set down as *the argument* of the non-contagionists, which, however, only proves that there are favorable and unfavorable conditions for its propagation. The same may be said to obtain in a greater of less degree in reference to all contagions, and if more striking in Cholera it only proves that it is peculiar in this as in many other respects.

In summing up the evidence in reference to the disease, furnished by a table of its prevalence in one part of the City of Chicago in the article so frequently referred to in the report before us, the inference was drawn that the period that elapsed between exposure and attack was generally one day. This statement is seized upon by the author of the report and the conclusion arrived at that "either the assumed time of incubation is a total error or the cases upon which the arguments for its primary importation and communicability are founded are totally irrelevant to the question." If a stated period of incubation had been determined and no reference to the necessity of a proper condition of the system for the action of the poison made; nor the fact that contagious formities may be imported referred to, the conclusion of the author might be a very good one; but as this is only a comment upon the result of the statistical table above referred to, and all of the other points were maintained in the article from which the extract is made, it is a very violent conclusion and shows the necessity of unfair means to make out an argument. This necessity is further corroborated by the fact that the extract from the article as given in the report, and from which the conclusion is drawn, is garbled and thus rendered absured in its meaning. The following is the extract, which is twice quoted in the report, taken from the middle of a paragraph of comments upon the "Table showing the number of those exposed—The time between exposure and attack, and the duration of the disease in those who died in three blocks in Chicago." It will be seen that such an extract is an unwarranted perversion of the meaning of the author. It is as follows: "The emigrants came by way of New York and Buffalo a few days before taken, where the disease was prevailing when they passed. It shows the length of time between exposure and attack to be generally about one day which strictly corresponds with the history which follows." What shows? Why according to this extract the emigrants coming by way of New York and Buffalo; when with the context no one could reasonably mistake its reference to the table immediately above the paragraph, from the middle of which these two sentences are taken.

Immediately after the second insertion of this extract the author congratulates himself upon having disposed of the doctrine of contagion, saying "Much more might be adduced had we time or space but we conceive it needless."

Whether the author of the report aimed at ascertaining the truth in regard to the question discussed, or at vanquishing by special pleading the advocates of the doctrine of contagion, his document is so worded as to convey the impression to the reader that he conceived the latter to answer his purpose best, for he has apparently hunted for weak points in the arguments and left unnoticed those which are inexplicable upon any other hypothesis.

That Cholera is produced by a specific and not a general cause, we think must be apparent to any one who has taken the pains to examine the history of its spread from place to place in its marches from India to California. That it is portable we think clearly established by its invariably following in those marches the channels of human intercourse; by its being introduced into isolated and remote settlements, as at Folly Island and almost innumerable instances that have occurred in our own country, many of which have occurred in Illinois; and that it is communicable from person to person we think clearly manifested by its extending from points of introduction, in most instances traceable, through direct channels of communication to adjacent neighborhoods. We have in vain sought for a satisfactory explanation of these historical facts by any other than the doctrine of communication. The only one that *appears* to answer the purpose is that of Prof. Mitchell of Philadelphia, attributing to cryptogami the poison, which amounts to little more than an attempt to explain the essential nature of the virus by which communication, or as he calls it, importation takes place.

These facts have been disregarded by the author of the report, while coincidences in particular cases showing that certain conditions are sometimes present, and instances where the disease did not spread, with points of dissimilarity between it and some other diseases, acknowledged to be contagious, from the sum and substance of his arguments. Now coincidencs are of no account to the argument unless they generally occur; that a disease does not spread in every case of exposure does nothing toward proving that it is not by contagion that it is propagated where it does prevail, and that it is dissimilar to other contagious diseases, only proves its specific character, and that it differs in those respects from them, leaving the general fact of its being diffused from county to county and from place to place by channels of direct communication undisturbed and unaccounted for.

The Report of Dr. Rouse is a short, sensible, statement of his high appreciation of the value of Chloroform in mitigating the pains of Parturition. He bears testimony to its safety and utility in the strongest terms of commendation.

